# The effect of transition training program on nurse interns engagement

**DOI:** 10.1186/s12912-025-04127-0

**Published:** 2025-12-12

**Authors:** Shereen S. Khalifa, Samia M. Adam, Azza E. Mohamed

**Affiliations:** Nursing Administration Department, Faculty of Nursing, Ain Shams University, Cairo, Egypt

**Keywords:** Engagement, Nurse interns, Transition

## Abstract

**Background:**

Nursing interns frequently experience transition shock. Transition is a complex and continuously shifting process that involves vital changes in roles, responsibilities, and goals as one phase ends and another begins.

**Aim:**

The purpose of the study was to investigate the effect of a transition training program on work engagement among nurse interns.

**Methods:**

Using a quasi-experimental design consisting of a single-group pretest, posttest, and follow-up test, the study was carried out at the Cairo, Egypt-affiliated Ain-Shams University Hospital, El-Demerdash Hospital, Pediatrics Hospital, and Cardiovascular Hospital. A simple random sampling technique was used, including 160 nurse interns. Data were gathered using three instruments: the nurse interns’ transition knowledge questionnaire, the Casey-Fink Graduate Nurse Experience Survey, and the Utrecht Work Engagement Scale.

**Results:**

The nurse interns’ transition knowledge, experience, and work engagement showed significant improvement in post-implantation training program. Post-training; the majority of nurse interns’ had satisfactory knowledge with some decline at follow-up phase. Also, the majority of nurse interns’ had satisfactory transition experience with some decline at follow up phase. Additionally, post training the most of nurse interns had high level of work engagement with some decline at follow-up phase.

**Conclusion:**

The knowledge, transition experience, and work engagement of nursing interns significantly improved following the implementation of the transition training program. To fill the gaps in clinical preparation, competency development, and the transition to professional practice, nursing education programs should implement structured internship curricula that combine academic knowledge with a variety of clinical experiences.

**Clinical trial number:**

Not applicable.

## Background

Nursing internships are an important part of nursing practice education and a critical time in nursing students’ development of their professional values [1]. Nursing interns have numerous challenges in clinical settings throughout their internship year, including the disconnect between their theoretical and practical knowledge [2]. A healthcare worker in training is known as a nursing intern. They provide direct patient care and perform a variety of clinical duties under the supervision of a registered nurse. Typically, nursing students seeking practical experience in a hospital or medical center are nurse interns [3].

As nurses make up the largest professional group in a healthcare organization, their contributions are often crucial to the standard and safety of the services provided [4]. Through the transition program, nursing interns can advance beyond their level of a bachelor’s degree and learn how to handle the rigors of complicated patient treatment. Consequently, transition programs provide new nurses with the means to better understand their duties and obligations concerning challenges in nursing practice that are ethical, legal, professional, and clinical [5]. To gain new knowledge, better techniques, and hone their abilities for greater engagement and improved operational performance, the training program has grown into a massive endeavor [6].

Transition is a complex and continuously shifting process that involves vital changes in roles, responsibilities, and goals as one phase ends and another begins [7]. Transition shock is a difficult and frightening process that involves a wide range of physical, emotional, psychological, and intellectual changes when recently graduated nurses take on a professional practice role for the first time [8]. Nursing interns who experience high levels of transition shock may find it difficult to adjust, which could negatively affect team cohesiveness and patient safety by increasing turnover rates and adverse occurrences [9]. According to research, nursing interns who experience less transition shock typically have greater levels of self-efficacy and core skills, which help them deal with clinical nursing challenges, adjust to clinical nursing practice, and support the development of their physical and mental health [10].

Organizations have been paying more attention to engagement in recent years because they believe that there is a connection between organizational success and employee engagement. One important sign of a successful and healthy company is employee engagement. Engagement encompasses involvement, dedication, zeal, fervor, integration, concentrated work, and strength. Additionally, engagement has been described as a consistent, widely distributed, and positive emotional motivational state of fulfillment in professionals and the utilization of members’ identities in their job positions [11].

Nurses who feel engaged in their work are more likely to be motivated to support organizational goals, feel a sense of community, and find fulfillment in their duties. Several factors, such as chances for career advancement, encouraging relationships with managers and colleagues, and compatibility with the organization’s principles, can increase nurses’ sense of embeddedness and, their level of work engagement [12].

Preceptorship is a common method in the United Kingdom to assist newly qualified nurses as they transition to professional practice. Preceptorship is defined by the Nursing and Midwifery Council (NMC) as a period of structured transition for newly registered practitioners, during which they are supported by an experienced practitioner to develop their confidence as an autonomous professional and to refine their skills, values, and behaviors [13, 14].

### Significance of the study

For nurse interns, the transition from student to professional nursing is a critical period in their development as professional nurses [15]. The newly graduated nurses’ transition experience is a difficult and demanding time of adaptation [16]. Transition shock can cause severe stress, worry, and tension, as well as separation symptoms, psychological anguish, and disengagement from work [17]. Additionally, theory-practice gaps are commonly reported reasons for new graduate nurses to leave nursing jobs [18].

Transition shock occurs when people are impacted by their roles, relationships, knowledge, and responsibilities during role and environmental transitions; they may sense and experience doubt, disbelief, disorientation, and uncertainty of orientation [19]. Nursing interns frequently experience transition shock [20]. Finally, the primary root of the transition problem is the shock that nursing students have when they face the real world and realize that their perception of the workplace varies from what they learned in academia. Help close the knowledge hole between academia and application. It is important to implement transition training programs for nurse interns to offer ongoing chances for practice and the application of critical knowledge to patient care.

### Study aim

The purpose of the study was to investigate the effect of a transition training program on work engagement among nurse interns. The study hypothesized that the application of the transition training program would increase nurse interns’ work engagement.

## Methods

### Study design

A quasi-experimental design of single-group pretest, posttest, and follow-up test was utilized in conducting study.

### Study settings

The study was carried out in the Ain-Shams University Hospitals affiliation, Cairo, Egypt, specifically the Pediatrics Hospital, Cardiovascular Hospital, El-Demerdash Hospital, and Ain-Shams University Hospital, where nursing interns carried out their training. Critical care units at these hospitals served as teaching grounds for nursing interns. Patients in various medical disciplines, heart and vascular conditions, and surgical specialties were treated at these facilities.

The internship year consisted of 12 months of clinical training at hospitals. Typically, the program starts with an orientation phase where interns are introduced to professional expectations, safety regulations, and institutional policies. Every two months after that, interns participate in clinical rotations where they visit different hospitals and departments, including pediatrics, emergency, maternity, medical-surgical, and critical care, to obtain practical experience under supervision. A final evaluation phase, which combines assessments, marks the end of the program. Accountability and organized learning are guaranteed by documentation in the form of logbooks, attendance logs, and reports. Each intern was assigned to a clinical nurse an approximate 1:1–3 intern-to-clinical nurse ratio depending on unit workload and patient acuity. Academic oversight was provided by a clinical faculty member for every 12–15 interns.

An internship program bridges the gap between student education and independent clinical practice. It is typically an organized, institutional phase that comes after academic coursework. Under both academic and clinical supervision, it emphasizes the broad integration of skills across various specialties [1]. Conversely, a preceptorship program places a strong emphasis on the one-on-one mentoring of a student or novice nurse during clinical placements or the early phases of employment by an experienced registered nurse. Role socialization, professional adjustment, and the development of clinical judgment within a particular unit or specialty are the goals of a preceptorship [13].

The case method of nursing care delivery was adapted in which nurse interns were responsible for providing comprehensive care to an assigned patient under direct supervision [21]. This approach allowed students to integrate theoretical knowledge with clinical practice, develop accountability and enhance critical thinking. In addition, clinical nurses supported the interns in daily patients care activities [22], while head nurses provide administrative guidance and ensure coordination with clinical units [23]. Faculty staff and their assistants play vital role in mentoring, supervising skill performance and effective constructive feedback. The internship orientation program was structured before starting the period of training and it consisted of varies sessions related to clinical practice. Additionally before each clinical round, students spent two weeks orientation about rules and policies of units.

Clinical nurses assist nursing students by orienting them to the clinical environment, routines, and policies; ensuring patient safety; and supporting the application of theoretical knowledge into clinical practice. They serve as professional role models, demonstrating ethical behavior, communication, teamwork, and time management. Clinical nurses also facilitate students’ participation in patient care under direct supervision, providing opportunities for skill development within the limits of safety and institutional policy. Additionally, they collaborate closely with faculty members to coordinate students’ clinical activities and provide feedback regarding performance, clinical competence, and professional conduct. Although formal evaluation remains the responsibility of the faculty member, clinical nurses contribute through informal feedback and continuous support that enhance students’ confidence and clinical learning [24–26].

### Study sampling and participants

Simple random sampling was employed. Simple random sampling was applied to select participants. A complete list of all eligible nursing interns enrolled during the academic year (2024–2025) was obtained, and each intern was assigned a unique identification number. Using a computer-generated random number table, the required sample was drawn without replacement. This ensured that every eligible intern had an equal chance of selection and reduced the risk of selection bias. The total number of nurse interns enrolled in Ain-Shams University Hospitals in the first six months of the internship year (2024–2025) was 272 interns. The sample size was 160 nurse interns, calculated based on [27].

$${\rm{n}} = {{{\rm{N}} \times {\rm{p}}\left( {1 - {\rm{p}}} \right)} \over {[{\rm{N}} - 1 \times \left( {{{\rm{d}}^2} \div {{\rm{Z}}^2}} \right] + {\rm{p}}\left( {1 - {\rm{p}}} \right)}}$$

*n* = sample size

*N* = total size

Z = 1.96

d = error level 5%

*p* = 0.5

### The inclusion criteria

Nurse interns who received training at Ain Shams University Hospitals throughout the first half of their internship year (2024–2025) were approved to take part in the study.

### The exclusion criteria

Nurse interns who were postponing and repeating their internship training from last year.

### Study concepts

1. An internship is a focused learning opportunity for students in a particular academic field. It serves as the foundation for knowledge application, skill development, and professional socialization, allowing students to transition from dependent supervised practice to independent collaborative practice [28].
2. An internship program is thought to be a successful strategy for giving college students general knowledge and experience. Internships, in short, become a way to gain independence and self-determination. Management, organization, and planning of the internship program are critical to its effectiveness [29].
3. Nursing interns are students who have completed a four-year Bachelor of Science in Nursing program [30].
4. The definition of engagement in professionals is a consistent, distributed, and positive emotional motivational state of fulfillment and the ability to fully commit to one’s work roles [11].
5. Nursing staff members’ work engagement is a communal phenomenon that is connected to a variety of concepts, including burnout, emotional commitment, organizational commitment, safety, empowerment, meaningfulness, organizational citizenship, and nursing job satisfaction [31].
6. A transition is a time of change that occurs in a person or an environment that shares specific characteristics. These are discrepancies between the expectations that were prevalent in the past and those that are prevalent in the present [32].
7. A training program is a planned, methodical collection of learning exercises intended to improve participants’ knowledge, abilities, and competencies in a particular field, typically in order to boost output, adjust to new responsibilities, or accomplish organizational objectives [33].
8. Transition programs are orientation courses that cover clinical skills, patient safety, evidence-based practice, and leadership in great detail. They were created to support nurses and nursing students as they move into clinical practice and enhance their retention by fostering the growth of their clinical and leadership abilities [34].
9. The term “transition knowledge” describes nurses’ comprehension, awareness, and proficiency with theoretical and practical subjects throughout a transitional era that allow them to adjust [5].

## Data collection

Three tools were used in this study, namely the Nurse Interns’ Transition Knowledge Questionnaire [35–39], the Casey-Fink Graduate Nurse Experience Survey [40], and the Utrecht Work Engagement Scale [41].

### Nurse interns’ transition knowledge questionnaire

This questionnaire consisted of two parts: (I) nurse interns’ job and personal characteristics. It included age, sex, marital status, training hospital, and attendance of training workshops; and (II) the Transition Knowledge Questionnaire was developed by researchers based on related literature [38–39]. It was aimed to assess nurse interns’ transition knowledge pre-, post-, and follow-up the program phases. It consisted of 40 multiple-choice questions (MCQs) such as transition shock can be defined as, examples of nonverbal communication in the work environment, and which of the following scenarios demonstrates critical thinking in nursing. It covered aspects of nurse interns’ transition knowledge, including transition shock, transition role, documentation, critical thinking, socialization, communication, evidence-based practice, and patient safety.

Each of the 40 knowledge questions was coded as 1 for a correct answer and 0 for an incorrect answer. In SPSS, we used Transform → Compute Variable to create the total score by summing all 40 items (possible range = 0–40). The percentage score was then calculated with the formula: (Total score/40) * 100. For each dimension, we calculated the mean score by adding the scores of its items and dividing by the number of items in that dimension, then converted it to a percentage. Finally, we used Transform → Recode into Different Variables to categorize participants: scores > 60% = satisfactory and scores < 60% = unsatisfactory [36]. The internal consistency and reliability of the tool for nurse interns’ transition knowledge was 0.88.

### Conceptual definition of the nurse interns’ transition knowledge questionnaire dimensions

1. Transition shock is defined as a difficult and frightening process that involves a wide range of changes in a freshly graduated registered nurse’s physical, emotional, developmental, and intellectual characteristics when they take on a professional practice role for the first time [8].
2. Role transition is any occurrence or non-occurrence that causes connections, routines, presumptions, and roles in the contexts of oneself, one’s job, one’s family, one’s health, and the economy to change. When a person transitions to a new event, they must be able to anticipate, understand, and accept the change [42].
3. Nursing documentation consists of written and printed forms that need to be filled out completely, properly, and flexibly in order to track patient care outcomes, gather crucial data, comply with regulatory requirements, and maintain quality control [43].
4. Critical thinking is a dynamic, intricate process made up of strategic abilities and attitudes that aims to accomplish a particular goal. Critical thinking is defined as being particular, systematic, and organized. It also entails asking questions to better comprehend what is happening and why, as well as being curious about the facts, motives, and justifications for a concept or action [44].
5. Nursing socialization is defined as themes of affirmation, knowing, and belonging [45].
6. Evidence-based practice is the combination of clinical knowledge, patient values, and the greatest available scientific findings [46].
7. Communication is the transfer of knowledge and comprehension from one individual to another. In order to modify behavior, communication involves both verbal and nonverbal exchanges between the sender and the recipient [47].
8. Patient safety is a major issue for healthcare systems around the globe. Improving healthcare outcomes requires making sure patients receive safe, high-quality care [48].

### The casey-fink graduate nurse experience survey

This survey was created by [40] and modified by researchers. It was aimed to assess nurse intern transition experience pre-, post-, and follow-up in the program phases. It consisted of 56 items divided into two sections, namely role transition experience (41 items) and learning needs assessment (15 items). Responses of nurse interns for Section I were measured on a four-point Likert scale ranging from strongly disagree (1), disagree (2), agree (3), and strongly agree (4). While responses of nurse interns for section II were measured on a three-point Likert scale ranging from not confident (0), somewhat confident (1), and highly confident (2). The mean score for the section was calculated by adding up the item scores for each dimension and dividing the total by the number of questions. Percentage scores were calculated from these mean values. The transition experience of the nursing interns was deemed satisfactory if it exceeded 60% and unsatisfactory if it fell below 60% [37]. The internal consistency and reliability of the Casey-Fink Graduate Nurse Experience Survey were 0.90.

### Utrecht work engagement scale

This scale was developed by [49] and adopted from [41]. This scale was aimed to assess nurse interns’ work engagement pre-, post-, and follow-up to the program phases. It consisted of 25 items divided into three dimensions namely, vigor (9 items), dedication (7 items), and absorption (9 items). A Likert scale with five possible answers—never (0), rarely (1), sometimes (2), often (3), and always (4)—was used to gauge the responses of nursing interns. A mean score was calculated for each dimension by adding together the item scores and dividing the total by the number of questions. Work engagement was categorized as moderate if the score fell between 35 and 68, higher if the score fell between 69 and 100, and lower if the score fell between 0 and 34 [41]. The internal consistency and reliability for the Utrecht Work Engagement Scale was 0.85.

### Intervention phases

Beginning in October 2024 and ending in June 2025, the study’s intervention ran for nine months. Three separate data collection methods were used during the study: pre-program, immediately post-program, and follow-up after three months of the program. Assessment, planning, implementation, evaluation, and follow-up were the stages that were included.

### Assessment phase

Using the appropriate data collection instruments, this phase pre-tested the study nurse interns’ transition experience, knowledge, and work engagement. Using the appropriate data collection instruments, this phase pre-tested the study nurse interns’ transition experience, knowledge, and work engagement. This phase lasted approximately one month until the end of October 2024, during which time the nurse interns who provided written consent to participate were given questionnaire sheets to evaluate their work engagement, transition knowledge, and transition experience, along with instructions on how to fill them out.

### Planning phase

Following the completion of the assessment phase’s data collection, an analysis was conducted to determine the transition knowledge and experience of the nurse interns’ strengths and limitations. It also included every statement that the researchers reported and documented. Until the end of November 2024, this process took about one month to complete. The researchers created the booklet and content for the transition training program based on the data gathered from the analysis of the assessment phase.

### Implementation phase

The general objective of this training program is to prepare the nurse interns to adapt to the transition smoothly. Mini lectures, group discussions, brainstorming, role-playing, small-group activities, and discussion participation are some of the teaching strategies that are employed. Videos, data displays for PowerPoint presentations, and handouts are examples of media employed. During each training program session, the researcher discussed with nurse interns the strong and weak tasks regarding the transition period. At the end of the training program, each nurse intern was handed the transition training program booklet. Also, after implementing the transition training program, the researcher held many meetings with nurse interns to discuss issues related to their performance and answer their questions, if any. The group meeting started every day from 11:00 am to 1:00 pm. This phase took two months until the end of February 2025. The training program consisted of eight sessions were as follows:

- Session 1: It was concerned with the signs and symptoms of transition shock, explaining factors contributing to transition shock, and how to utilize coping strategies and support systems.
- Session 2: It was covered the communication among caregivers, learn techniques for clear and professional communication, identify barriers to effective nurse-to-nurse communication, and Practice real-life communication scenarios.
- Session 3: It was concerned with principles of proper documentation among nurses, explaining common documentation errors and how to avoid them, and demonstrated correct documentation techniques using real scenarios.
- Session 4: It covered the importance of critical thinking in clinical decision-making and applying critical thinking strategies in nursing.
- Session 5: It was concerned with Evidence Based Practice “EBP”, how to apply the process of EBP, and how to utilize strategies for overcoming EBP barriers.
- Session 6: It was related to identifying roles and responsibilities of a nurse intern, understanding challenges in transitioning to a professional role, and explaining strategies to adapt to the transition smoothly.
- Session 7: It was concerned with the stages of socialization in nursing, and identify common challenges faced by nurse interns during socialization and explore strategies to enhance professional adaptation.
- Session 8: It covered the patient’s safety, identify who is responsible for ensuring patient safety, and explain methods and techniques of patient safety.

### Evaluation phase

The researcher assessed the intervention’s impact on nurse interns’ transition knowledge, transition experience, and work engagement as soon as the transition training program was put into place. The same data collection instruments used in the assessment phase were used for this. This stage lasted for a month.

### Follow-up phase

Three months following the post-assessment evaluation, the same procedure was carried out again for follow-up, utilizing the same data collection instruments. This stage lasted for a month.

### Ethical considerations

We confirm that our study was conducted in accordance with the principles outlined in the Declaration of Helsinki. The Research Ethics Committee of the Faculty of Nursing at Ain Shams University in Cairo, Egypt gave its approval to the study [code number: 25.02.570]. The researchers met nurse interns and introduced themselves to explain the aim of the study and its objectives, ways to fill in the different tools, and sought their approval to participate and cooperate in the study throughout the training program phases. Written consent was taken for nurse interns who participated in the study. In addition to being informed of their freedom to withdraw from the study at any time and without explanation, nurse interns were reassured that any information collected would be kept private and utilized exclusively for research. Students were informed that their decision would not affect their evaluation or relationship with faculty of nursing, faculty staff,or their assistants. Data collection was conducted in non-teaching context to avoid perception of coercion. The researcher ensured anonymity and confidentiality of nurse interns by kept responses with codes rather than names used in data analysis.

### Statistical analysis

The statistical software program SPSS 2023 was used for data entry and statistical analysis. Frequency and percentages for qualitative variables are used to display the data. The study employed multiple linear regression analysis, analysis of variance (ANOVA) for the full regression models, and the chi-square and Cochran’s Q tests to compare the qualitative variables and investigate the associations between two qualitative variables. Using Pearson’s rank correlation, the associations between the ranked and quantitative variables were evaluated. To evaluate the produced tool’s internal consistency and dependability, the Cronbach alpha coefficient was computed. At a p-value < 0.05, statistical significance was established. The p-value was less than 0.001, which indicated high statistical significance.

## Results

### Nurse interns’ personal and job characteristics

Table 1 indicates that 86.3% of interns were aged ≥22 years old, 59.4% were female, and 79.4% of them were single. Also, 32.5% of them had clinical training at Ain-Shams University Hospital. Additionally, 90.6% of nurse interns didn’t attend training workshops, and only 9.4% of them attended training courses.

**Table 1:** Nurse interns’ personal and job characteristics (*n* = 160)

Personnel and Job Characteristics	(N)	(%)
**Age (year)**		
< 22	22	13.8
≥22	138	86.3
Mean±SD	22 ± 0.483	
**Sex**		
Male	65	40.6
Female	95	59.4
**Marital Status**		
Single	127	79.4
Married	33	20.6
**Training Hospital**		
Ain-Shams University Hospital	52	32.5
El-Demerdash Hospital	43	26.9
Pediatrics Hospital	30	18.8
Cardiovascular Hospital	35	21.9
**Attended training workshops**		
Yes	15	9.4%
No	145	90.6%

### Nurse interns’ transition knowledge throughout the program phases

Table 2 indicates that 78.1% of nurse interns had unsatisfactory levels of total knowledge at the pre-intervention phase. In contrast, between 93.8% and 80.6% of them completed the post-follow-up intervention phase with a satisfactory level of overall knowledge. Additionally, at a *p* value (*p* < 0.00), improvements were statistically significant during the post-intervention and follow-up phases.

**Table 2:** Comparison between total nurse interns’ transition knowledge throughout the program phases (*n* = 160)

Total transition knowledge	Pre		Post		FU		Cochran’s Q	P value
	N	%	N	%	N	%		
Satisfactory knowledge (60 ≥)	35	21.9	150	93.8	129	80.6	185.9	0.000**
Unsatisfactory knowledge ( < 60)	125	78.1	10	6.3	31	19.4		

### Nurse interns’ transition experience throughout the program phases

Table 3 concludes that 68.1% of nurse interns had satisfactory experience regarding the stress/burnout dimension at the pre-intervention phase. While 96.9%-90% of them were satisfactory experience regarding the organize/prioritize care dimension at post follow-up intervention phase. A *p* value (*p* < 0.00) indicated that the differences were statistically significant.

**Table 3:** Nurse interns’ transition experience dimensions throughout the program phases (*n* = 160)

Transition experience dimensions	Satisfactory experience (60 ≥)						(Pre-Post)		(Pre-Fu)	
	Pre		Post		FU					
	N	%	N	%	N	%	χ2	P value	χ2	P value
Section I: Role transition experience	57	35.6	151	94.4	140	87.5	18.7	0.00**	11.6	0.00**
Section II: Learning needs assessment of skills	38	23.8	142	88.8	135	84.4	7.7	0.00**	9.2	0.00**

Table 4 shows that 29.4% of nurse interns had satisfactory transition experience at the pre-intervention phase. While 91.3% of them had a satisfactory transition experience post-intervention. Also, there was a decline to 84.4% at the follow-up intervention. Additionally, improvements in the post-intervention and follow-up phases were statistically significant.

**Table 4:** Comparison between total nurse interns’ transition experience throughout the program phases (*n* = 160)

Total transition experience	Pre		Post		FU		Cochran’s Q	P value
	N	%	N	%	N	%		
Satisfactory experience (60 ≥)	47	29.4	146	91.3	135	84.4	141.3	0.000**
Unsatisfactory experience ( < 60)	113	70.6	14	8.8	25	15.6		

### Work engagement levels among nurse interns throughout the program phases

Table 5 indicates that (6.2%) of nurse interns had high levels of work engagement regarding the dedication dimension at pre-intervention phase. While the majority (93.8%- 90%) of them had high levels of work engagement regarding the vigor dimension at the post-follow-up intervention phases. The differences were statistically significant with a *p* value (*p* < 0.00).

**Table 5:** Nurse interns’ work engagement dimensions throughout the program phases (*n* = 160)

Work engagement dimensions	High level of work engagement						(Pre-Post)		(Pre-Fu)	
	Pre		Post		FU					
	N	%	N	%	N	%	χ2	P value	χ2	P value
Vigor	7	4.3	**150**	**93.8**	**144**	**90**	118.4	0.00**	84.6	0.00**
Dedication	**10**	**6.2**	147	91.9	133	83.1	123.3	0.00**	124.8	0.00**
Absorption	4	2.5	145	90.6	136	85	125.9	0.00**	65.1	0.00**

Table 6 demonstrates that during the pre-intervention phase, 60% of nursing interns showed low levels of work engagement. Furthermore, throughout the post-intervention phase, 90.6% of them showed high levels of work engagement. 85.6% of them also demonstrated high levels of work engagement during the follow-up intervention.

**Table 6:** Comparison between total nurse interns’ work engagement levels throughout the program phases (*n* = 160)

Total nurse interns’ work engagement levels	Pre		Post		FU		(Pre-Post)	(Pre-Fu)	P value
	N	%	N	%	N	%	χ2	χ2	
High	7	4.4	145	90.6	137	85.6			0.000**
Moderate	57	35.6	13	8.1	18	11.3			
Low	96	60.0	2	1.3	5	3.1	24.1	21.3	

### Correlation between the overall transition experience, knowledge, and work engagement of nursing interns during intervention phases

Through intervention phases, Table 7 shows that the total transition knowledge, total transition experience, and total work engagement of nursing interns were statistically significantly positively correlated (*p* = 0.00).

**Table 7:** Correlation between total nurse interns’ transition knowledge, transition experience, and work engagement through intervention phases

Intervention phases		Total transition knowledge		
		Pre	Post	FU
Total nurse interns’ transition experience	R	0.48	0.37	0.45
	P	0.000**	0.000**	0.000**
Total work engagement	R	0 0.69	0.59	0.44
	P	0.000**	0.000**	0.000**

Intervention phases		Total transition experience		
		Pre	Post	FU
Total work engagement	R	0.54	0.35	0.59
	P	0.000**	0.000**	0.000**

## Discussion

Nurses are essential in advancing medical treatment and health equity. As advocates for patients in a variety of healthcare settings, nurses can collaborate with other medical professionals to advance health equity for all patients [50]. Transitioning from a student to a professional nursing career is a challenging journey [15, 51]. The study aim was to investigate the effect of a transition training program on work engagement among nurse interns.

### Nurse interns’ transition knowledge throughout the program phases

In light of the total nurse interns’ transition knowledge throughout the program phases. The current research indicated that over three-quarters of nurse interns had an unsatisfactory level of total knowledge at the pre-intervention phase. But at the post-follow-up intervention phase, the majority of them had a satisfactory level of overall knowledge.

Unsatisfactory knowledge before applying the training program regarding transition, which could be because of inadequate clinical orientation before the internship year. To enhance nurse clinical learning and close the theory-practice gap, transition training programs must be incorporated into the curriculum. The program is more comprehensive regarding the topics most practiced in the hospital during the internship year, therefore an impact on knowledge was found after the program. According to similar results, the experimental group demonstrated a significant improvement in general clinical nursing skills, ethics, overall assessment, work self-efficacy, and decreased occupational stress when the intervention was implemented [52]. Consistent with [36], who stated that following the introduction of the transition training program, the majority of nurses possessed satisfactory transition knowledge.

Also [53], demonstrated that the transition training program works well for fostering trained nurses’ confidence, engagement, and acquisition and retention of knowledge. Additionally [54], presented that oriented transition programs with sufficient practice opportunities in clinical sites result in improved clinical knowledge and competencies in new graduate nurses.

### Nurse interns’ transition experience throughout the program phases

As regards nurse interns’ transition experience dimensions throughout the program phases. According to the results of the study, two-thirds of nursing interns had satisfactory experiences with stress aspects before intervention. While most of them had satisfactory experience regarding organization/prioritization of care dimensions at the post-follow-up intervention phase. Stress prior to training programs could be attributed to difficulties setting priorities and efficiently managing time in hectic settings.

These results are aligned with [55], which revealed that stress affected over half of the nurses. Also, these findings agree with [56], who presented that most participants agreed regarding experiencing stress in their personal lives because of work. [57], who concluded that most of the participants expressed stressful, ambiguous, and overwhelming feelings during the transition period. Additionally [58], consistent with study findings, it showed that during role transition, over half of recent grads felt stressed.

To total the nurse interns’ transition experience. According to the study, less than one-third of nursing interns had a satisfactory pre-intervention transition experience. While most of them had a satisfactory transition experience at the post-intervention. With the follow-up intervention declining as well. Potential expositions may be that transition programs help nurses adjust to the pace, culture, and expectations of the healthcare setting. Acquired knowledge reflecting confidence in clinical skills and practice, leading to a satisfactory transition experience during the internship year. Some decline in follow-up phase may be due to increased workload, and not applying preceptorship in clinical units.

Similar findings were reported by [5], who reported that following the implementation of a training program, the transition experience improved significantly. Consistent with [36] reported that most nurses had satisfactory transition roles when the transition training program was put into place.

### Nurse interns’ work engagement levels throughout the program phases

Concerning nurse interns’ work engagement dimensions throughout the program phases. At the post-follow-up intervention period, the study revealed that the majority of them had high levels of work engagement with relation to the vigor dimension. Furthermore, there were some declines during the follow-up stages, but statistically significant improvements during the post-intervention phases. There was statistical significance in the discrepancies.

A possible explanation is that conducting a training program makes nurse interns more likely to feel motivated if they believe they have control over their responsibilities. Engagement decreased at follow up phase likely due to reduce reinforcement in clinical areas. Additionally, improving congruence with the organization’s values and a feeling of purpose. In the same line as the present study [37], who revealed that high levels regarding the vigor dimension.

In light of the total nurse interns’ work engagement levels throughout the program phases. At the pre-intervention stage, less than two-thirds of nursing interns reported low levels of work engagement. While the majority of them had high levels of work engagement at the post-intervention phase. Also, most of them had high levels of work engagement at the follow-up intervention.

This could be due to nurse interns facing transition shock and may lead create a negative professional self-perception, which can lead to a lack of motivation at work. The organized learning environment, frequent feedback, and encouraging supervision offered during the training are all responsible for the increase in engagement that occurred after the program. Interns’ confidence, sense of competence, and motivation were all boosted by these factors, and their sense of belonging was strengthened by peer interaction and faculty support, all of which raised engagement levels.

In the same line as the present study [36], presented high levels of work engagement after conducting training knowledge regarding the transition role. Similar findings were reported by [58] that negative correlation between transition shock, work engagement, and enhancement by clinical training. Additionally [8], who indicated that there was improvement in work engagement at the intervention phases.

### Relationship between the overall transition experience, knowledge, and work engagement of nursing interns during intervention phases

The present investigation indicated that the overall transition experience of nursing interns with transition knowledge and overall work engagement showed statistically significant positive correlations. There was a significant improvement after conducting the transition training program. Potential expositions may be the transition training program helping interns clearly define their roles and responsibilities during the internship year, and accept their new working environment and understand expectations, so this helped smooth the transition period.

In keeping with the study [37], who presented significant correlations between nurse transition role experience with transition knowledge, and work engagement. Similar findings were reported by [59] who concluded that work engagement during transition shock and improvement during clinical training are negatively correlated. Also [60], who presented that residency programs facilitate work engagement.

While the findings of this study provide valuable insights into the effect of the transition training program on nurse interns’ engagement, several limitations should be acknowledged. First, the use of self-reported questionnaires may have introduced response bias, as participants’ answers could have been influenced by social desirability or recall bias. Second, the relatively short follow-up period limits the ability to assess the long-term effects of the training program on interns’ engagement. Third, the absence of a control group makes it more difficult to establish causal relationships between the intervention and the observed outcomes. Finally, although anonymity and confidentiality were ensured to all participants, the possibility of reaction bias cannot be completely ruled out.

## Conclusion

Based on the study’s findings, nursing interns’ knowledge during transition and level of work engagement during the internship year were both enhanced by the implementation of a transition training program. The results showed that following the implementation of the transition training program, the knowledge, transition experience, and work engagement level of the nursing interns improved in a highly statistically significant manner.

## Implications for practice and future directions

To fill gaps in clinical preparation, competency development, and the transition to professional practice, nursing education programs should implement structured internship curricula that combine academic knowledge with a variety of clinical experiences. Effective preceptorship applications are also essential for promoting competency development and professional advancement. Simulated emergency scenario training should be conducted during the orientation program. During the orientation program, it is crucial to create a work description for intern nurses and explain the program. Finally, a smooth transition from academia to practice can be promoted by promoting cooperation between nursing education programs and medical facilities.

## Data Availability

Due to confidentiality concerns, the data and materials used in the current study cannot be made publicly available. However, they are available from the corresponding author upon reasonable request.
